# Transcription Factors That Regulate the Pathogenesis of Ulcerative Colitis

**DOI:** 10.1155/2020/7402657

**Published:** 2020-08-24

**Authors:** Bing Zhang, Tao Sun

**Affiliations:** ^1^TCM Department, Hangzhou First People's Hospital, China; ^2^Hepatology Department, The Second Affiliated Hospital of Zhejiang Chinese Medical University, 318th Chaowang Road, Gongshu District, Zhejiang, 310005 Hangzhou, China

## Abstract

Ulcerative colitis (UC) is one of the inflammatory bowel diseases (IBD) characterized by occurrence in the rectum and sigmoid colon of young adults. However, the functional roles of transcription factors (TFs) and their regulating target genes and pathways are not fully known in ulcerative colitis (UC). In this study, we collected gene expression data to identify differentially expressed TFs (DETFs). We found that differentially expressed genes (DEGs) were significantly enriched in the target genes of *HOXA2*, *IKZF1*, *KLF2*, *XBP1*, *EGR2*, *ETV7*, *BACH2*, *CBFA2T3*, *HLF*, and *NFE2*. TFs including *BACH2*, *CBFA2T3*, *EGR2*, *ETV7*, *NFE2*, and *XBP1*, and their target genes were significantly enriched in signaling by interleukins. BACH2 target genes were enriched in estrogen receptor- (ESR-) mediated signaling and nongenomic estrogen signaling. Furthermore, to clarify the functional roles of immune cells on the UC pathogenesis, we estimated the immune cell proportions in all the samples. The accumulated effector CD8 and reduced proportion of naïve CD4 might be responsible for the adaptive immune response in UC. The accumulation of plasma in UC might be associated with increased gut permeability. In summary, we present a systematic study of the TFs by analyzing the DETFs, their regulating target genes and pathways, and immune cells. These findings might improve our understanding of the TFs in the pathogenesis of UC.

## 1. Introduction

Ulcerative colitis (UC) is one of the inflammatory bowel diseases (IBD) with symptoms such as abdominal pain, fever, malnourishment, fatigue, and weight loss [1]. UC is characterized by occurrence in the rectum and sigmoid colon of young adults aged 20-40 [2]. Currently, UC is recognized to be caused by the damages of the intestinal mucosal barrier and neuroendocrine and immune dysfunction due to the interplay of genetics, environment, and psychology [3], but its specific etiology and pathogenesis are still unclear.

With the advances in high-throughput technologies, a growing number of studies have been carried out to investigate the expression of some genes and proteins in the pathogenesis and molecular mechanism of UC. Specifically, the copy number variations (CNVs) in mitochondrial DNA have been identified as the predictor of UC-associated colorectal x`cancer by CNV arrays [4]. Moreover, FAM217B, KIAA1614, and RIBC2 were found to be hypermethylated in UC and could be used for the diagnosis and therapeutic treatment of UC based on genome-wide DNA methylation approach [5]. Furthermore, transcriptome-based system biology approach identifies ANP32E, a protein involved in steroid-refractoriness, indicating the key role of steroid-induced transcriptional changes and the implication of ANP32E in UC [6]. In addition to these genes or proteins, miRNAs have been found to be implicated in UC. Particularly, IL-33 expression was exerted by miR-378a-3p in an inflammatory environment, and downregulation of miR-378a-3p could result in IL-33 overexpression in UC [7]. These studies greatly improved our understanding of the underlying mechanism of UC pathogenesis.

In addition, the transcription factors (TFs), a series of molecules involved in regulating gene expression, have been emerged as key regulators in several diseases [8, 9]. Heat shock transcription factor 2 could predict mucosal healing and promote mucosal repair by suppressing MAPK signaling and inhibit intestinal epithelial cell apoptosis in UC through the mitochondrial pathway [10, 11]. Moreover, RUNX3 is also associated with UC by regulating the immune-related target genes and pathways [12, 13]. However, there is a lack of systematic study analyzing the functional roles of TFs in the pathogenesis of UC. Therefore, we carried out the present study, aiming at identifying the critical TFs, their downstream target genes, and pathways involved in UC pathogenesis.

## 2. Materials and Methods

### 2.1. Datasets

The gene expression data were collected from the Gene Expression Omnibus (GEO) database with accession GSE128682, and the sample collection was described in an earlier study [14]. The counts for each sample were normalized by DESeq2 [15]. The pairs of transcription factor (TF) target genes were downloaded from three public databases including JASPAR [16], TRANSFAC [17], and CHEA [18].

### 2.2. Differential Expression Analysis

The count-based expression data was used for the differential expression analysis (DEA). R/Bioconductor DESeq2 [15] was employed to identify the differentially expressed genes (DEGs). The two-fold change and adjusted *p* value of 0.05 were used to determine the DEGs for each comparison.

### 2.3. Transcription Factor Target Genes and Pathway Enrichment Analysis

The Fisher's exact test was used to identify the transcription factors (TFs) and pathways enriched by the DEGs. The DEGs with a significant correlation with their TFs were selected for this analysis and TFs with a large number of target genes (*n* > 2000) were excluded in the enrichment analysis. The enrichment analysis was implemented in the R clusterProfiler package with enricher function [19].

### 2.4. Immune Cell Proportion Estimation

The immune cell proportion was estimated by CIBERSORT, which used the gene expression profiles and immune cell-specific genes to characterize the cell composition of complex tissues [20]. The count-based expression data was normalized to Transcript Per Million (TPM) by R scater package (https://bioconductor.riken.jp/packages/3.4/bioc/html/scater.html), which was used for the CIBERSORT analysis.

### 2.5. Statistical Analysis

The two-sample comparison was tested by Wilcoxon rank-sum test or *t* test, and multiple-sample comparison was tested by analysis of variance (ANOVA). The Spearman correlation analysis was used to evaluate the correlation of two variables. Symbols of ∗, ∗∗, ∗∗∗, and ∗∗∗∗ indicate the statistical significances of 0.05, 0.01, 0.001, and 0.0001, respectively.

## 3. Results

### 3.1. Identification of Differentially Expressed Transcription Factors

**Figure 1:**
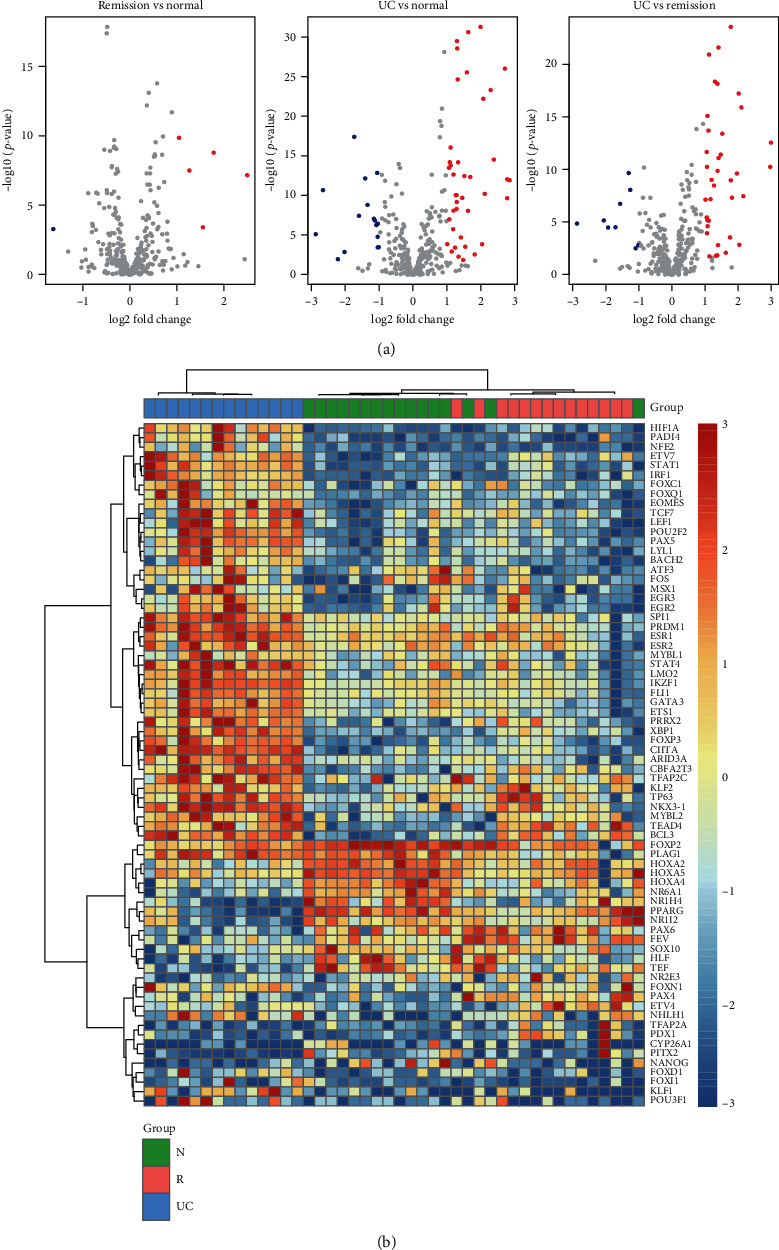
Differentially expressed transcription factors (DETFs). (a) The differential expression levels of the TFs. The X and Y axes represent the log2 fold change and -log10 (adjusted *p* value), respectively. (b) The gene expression profiles of the DETFs in UC, R, and N groups. The red and blue colors represent the high and low expression.

The mucosal biopsies had 14 ulcerative colitis (UC), 14 remission (R), and 16 healthy controls (N). With the three groups of mucosal biopsies, we compared one with the other two groups, respectively. UC had significantly different expression profiles as compared with R and N groups, with 3,202 and 2,517 differentially expressed genes (DEGs) in UC vs. N and UC vs. R (Supplementary Table S1), respectively. The comparison of R vs. N only identified 1,133 DEGs. Consistently, the comparisons of UC vs. N (*n* = 56) and UC vs. R (*n* = 46) had greater numbers of differentially expressed transcription factors (TFs) than that of R vs. N (*n* = 6) (Figure 1(a)). These results indicated that the transcriptomic profiles were significantly altered in UC samples as compared with samples of remission and healthy controls.

Totally, we identified 72 TFs significantly differentially expressed between the three groups (Supplementary Table S2). The hierarchical clustering analysis revealed that the UC samples could be clearly differentiated from the N and R samples by the TFs specifically upregulated in UC (Figure 1(b)). The TFs specifically upregulated in R and N samples also had the capability of classifying the two groups to some extent (Figure 1(b)). These results indicated that the TFs might be implicated in UC pathogenesis.

### 3.2. Expression Patterns of the Differentially Expressed Transcription Factors

**Figure 2:**
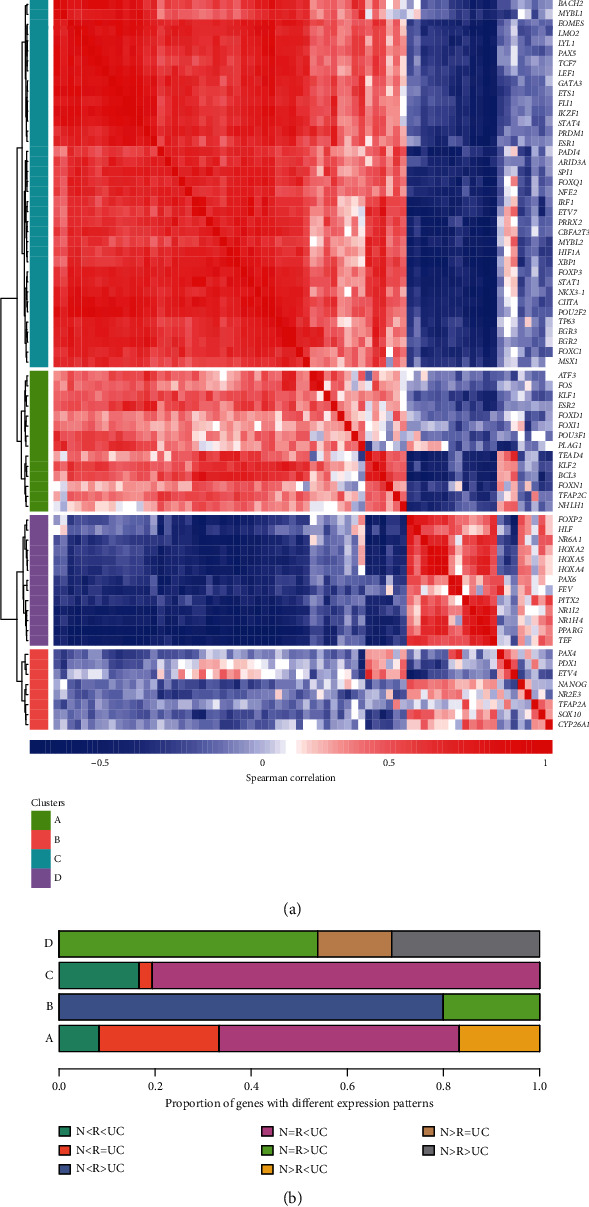
Gene expression patterns of the DETFs. (a) The coexpression modules of the DETFs. The modules were identified by the hierarchical clustering analysis with four clusters. (b) The proportion of DETFs in the eight gene expression patterns.

To reveal the expression patterns of the differentially expressed transcription factors (DETFs), we conducted coexpression analysis of the 72 DETFs. Notably, four categories of DETFs (A, B, C, and D) could be identified by the coexpression analysis (Figure 2(a)). Further analysis of the expression patterns revealed that upregulated TFs in UC (N = R < UC) were highly enriched in groups A and C, upregulated TFs in R (N < R > UC) had higher proportion in group B, and downregulated TFs in UC (N = R > UC) were more frequently observed in group D (Figure 2(b)). These results indicated that three categories were observed in these DETFs.

### 3.3. Target Genes of the DETFs

**Figure 3:**
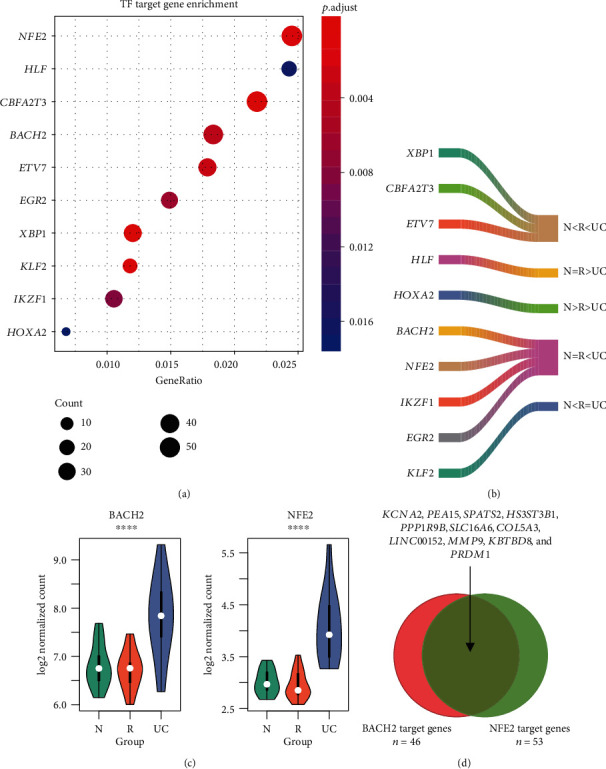
The DETFs with significant consequences of their target genes. (a) The DETFs significantly enriched by the DEGs. The node color represents the statistical significance calculated by Fisher's exact test. The node size represents the number of target genes with differential expression. (b) The expression patterns of nine DETFs significantly enriched by the DEGs. (c) The expression levels of BACH2 and NFE2 in the three groups. (d) The shared target genes between BACH2 and NFE2.

As the TFs could promote or suppress the transcription of their target genes, we then investigated whether the target genes were also differentially expressed. Specifically, DEGs were significantly enriched in the target genes of *HOXA2*, *IKZF1*, *KLF2*, *XBP1*, *EGR2*, *ETV7*, *BACH2*, *CBFA2T3*, *HLF*, and *NFE2* (Figure 3(a), Supplementary Table S3). Remarkably, *BACH2*, *NFE2*, *IKZF1*, *EGR2*, *XBP1*, *CBFA2T3*, and *ETV7* were upregulated in UC (N = R < UC or N < R < UC), and *HLF* and *HOXA2* were downregulated in UC (N = R > UC or N > R > UC) (Figure 3(b)). It should be noted that *BACH2* and *NFE2* were upregulated in UC (Figure 3(c)), and they had significantly more shared target genes (Figure 3(d)), suggesting that the two TFs might cooperate with each other to regulate their target genes.

### 3.4. Signaling Pathways That the DETFs and Target Genes May Participate in

**Figure 4:**
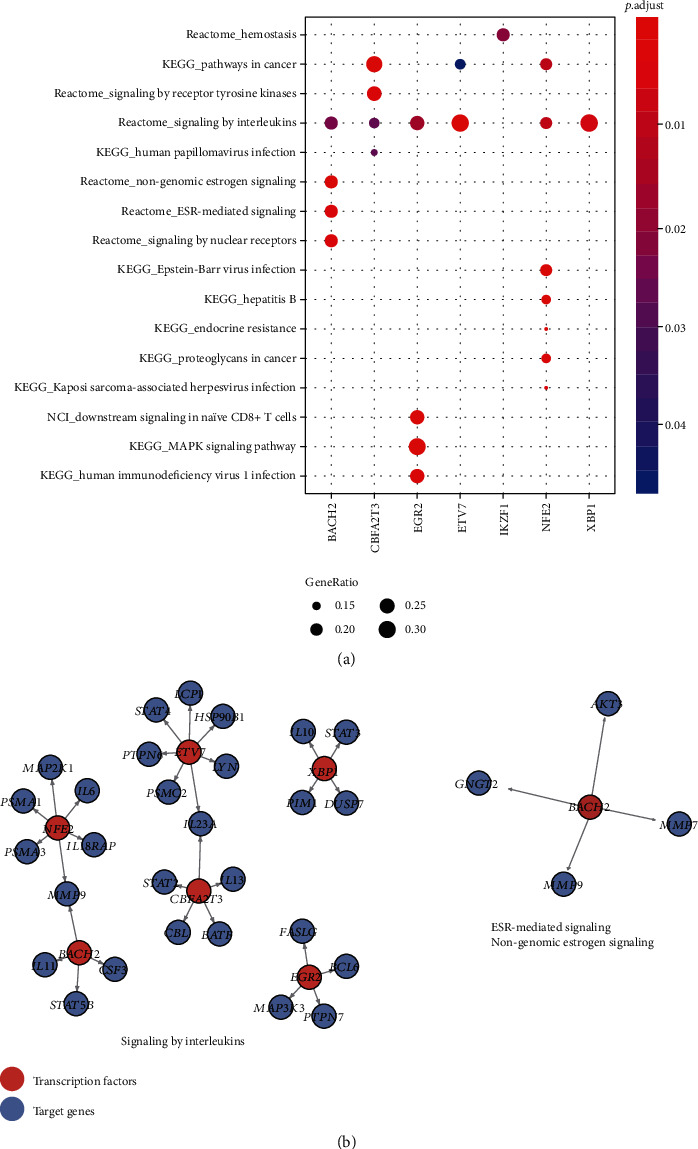
The signaling pathways enriched by the target genes of DETFs with differential expression. (a) The DETFs and their regulating signaling pathways. (b) The DETF-target pairs in signaling by interleukins, ESR-mediated signaling, and nongenomic estrogen signaling.

To further identify the signaling pathways regulated by the DETFs and target genes, we conducted a gene set enrichment analysis of the differentially expressed target genes of DETFs. We found that target genes of *BACH2*, *CBFA2T3*, *EGR2*, *ETV7*, *IKZF1*, *NFE2*, and *XBP1* were significantly enriched in the pathways (Figure 4(a)). The virus infection pathways including human papillomavirus infection, Epstein-Barr virus infection, Hepatitis B, Kaposi sarcoma-associated herpesvirus infection, human immunodeficiency virus 1 infection, and immune-related pathways such as downstream signaling in naïve CD8+ T cells and signaling by interleukins were significantly enriched by these target genes (Figure 4(a)).

Particularly, TFs including *BACH2*, *CBFA2T3*, *EGR2*, *ETV7*, *NFE2*, and *XBP1*, and their target genes were significantly enriched in signaling by interleukins. The inflammatory factors such as *IL6*, *IL18RAP*, *IL11*, *STAT5B*, and *CSF3* were involved in the signaling by interleukins (Figure 4(b)). Furthermore, target genes of *BACH2*, including *AKT3*, *GNGT2*, *MMP7*, and *MMP9*, were involved in ESR-mediated signaling and nongenomic estrogen signaling. These results indicated that estrogen signaling and signaling by interleukins might be closely associated with the UC pathogenesis.

### 3.5. Immune Cells and Their Association with DETFs

**Figure 5:**
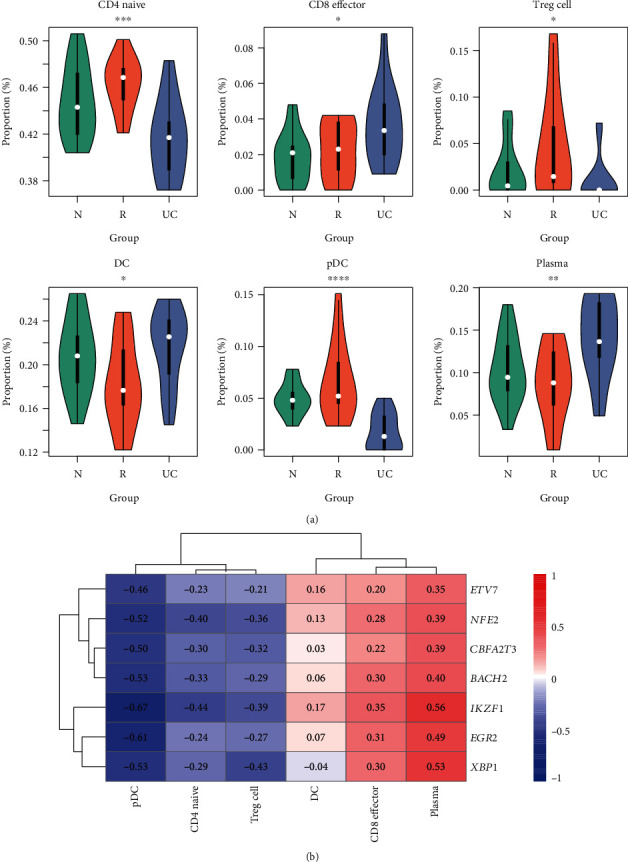
The association of transcription factors (TFs) with immune cell proportions. (a) The proportion of immune cells that were significantly accumulated or reduced in UC. (b) The Spearman correlation of TFs and immune cell proportions. The red and blue colors represent the positive and negative correlation.

As the inflammatory factors and pathways were potentially involved in UC pathogenesis, we investigated the relative abundance of immune cells in mucosal biopsies and their association with DETFs. The proportion of immune cells was estimated by CIBERSORT based on the gene expression profiles. Specifically, proportions of naïve CD4, regulatory T cells (Tregs), and plasmacytoid dendritic cells (pDC) were decreased in UC, while effector CD8 and plasma were increased in UC compared with R and N groups (Figure 5(a)). Particularly, DC was found to be reduced in the R group (Figure 5(a)). The correlation analysis revealed that the nine DETFs with functional enrichment of pathways including *BACH2*, *CBFA2T3*, *EGR2*, *ETV7*, *NFE2*, and *XBP1* were highly correlated with effector CD8 and plasma (Figure 5(b)), indicating that these TFs might promote the infiltration of effector CD8 and plasma into the intestinal mucosal tissues.

## 4. Discussion

Transcription factors (TFs) are key proteins involved in regulating gene transcription in cells. However, the functional roles of TFs and their regulating target genes and pathways are still little known in ulcerative colitis (UC).

In the present study, we collected gene expression data of mucosal biopsies from 14 UC, 14 remission (R), and 16 healthy controls (N), and identified DEGs in the three groups, of which, 72 were identified as differentially expressed TFs (DETFs). Furthermore, the coexpression analysis of the DETFs revealed three categories of TFs, which were upregulated in UC (N = R < UC), upregulated in R (N < R > UC), and downregulated in UC (N = R > UC).

As the function of DETFs could result in dysregulation of their target genes, we found that DEGs were significantly enriched in the target genes of *HOXA2*, *IKZF1*, *KLF2*, *XBP1*, *EGR2*, *ETV7*, *BACH2*, *CBFA2T3*, *HLF*, and *NFE2*. As BACH2 and NFE2 proteins had similar protein structure [21], they had a greater number of shared target genes. BACH2 has interactions with NFE2L1 and NFE2L3 based on BIOGRID [22] protein-protein interaction (PPI), indicating that BACH2 might also have the potential to interact with NFE2. Both BACH2 and NFE2 were implicated in UC via regulating inflammation-related pathways [23, 24].

Among the TF target genes, inflammatory factors such as *IL6*, *IL18RAP*, *IL11*, *STAT5B*, and *CSF3* were involved in the signaling by interleukins. The interleukins and receptors were frequently reported to promote the inflammatory phenotype in UC [25–28]. Notably, *IL11* and *IL18RAP* were identified as susceptibility loci in UC [29, 30]. Furthermore, target genes of *BACH2*, including *AKT3*, *GNGT2*, *MMP7*, and *MMP9*, were involved in ESR-mediated signaling and nongenomic estrogen signaling. As patients with UC have a higher risk for colorectal carcinoma (CRC) development [31] and the estrogen receptors (ER) alpha/beta balance has a relevant influence on colorectal carcinogenesis [32], we then speculated that the dysregulation of estrogen signaling might be associated with the risk of colorectal carcinogenesis.

Furthermore, to clarify the functional roles of immune cells on the UC pathogenesis, we estimated the immune cell proportions in all the samples. The accumulated effector CD8 and reduced proportion of naïve CD4 might be responsible for the adaptive immune response in UC, showing consistency with the previous study [33]. Notably, BACH2 and EGR2 could regulate CD8 cell differentiation, indicating that the high proportion of CD8^+^ might be associated with the upregulation of BACH2 and EGR2 [34, 35]. The accumulation of plasma in UC might be associated with increased gut permeability [36].

In summary, we present a systematic study of the TFs by analyzing the DETFs, their regulating target genes and pathways, and immune cells. These findings might improve our understanding of the TFs in the pathogenesis of UC.

## Data Availability

The gene expression data were collected from the Gene Expression Omnibus (GEO) database with accession GSE128682.

## Supplementary Materials

Supplementary 1 . Supplementary Table S1: UC had significantly different expression profiles as compared with R and N groups.

Supplementary 2 . Supplementary Table S2: 72 TFs significantly differentially expressed between the three groups.

Supplementary 3 . Supplementary Table S3: DEGs were significantly enriched in the target genes.
